# Supplementary figures and images for: Ordered, Random, Monotonic and Non-Monotonic Digital Nanodot Gradients
Source: PLoS One. 2014 Sep 5;9(9):e106541. doi: 10.1371/journal.pone.0106541 (PMC4156346; doi:10.1371/journal.pone.0106541)

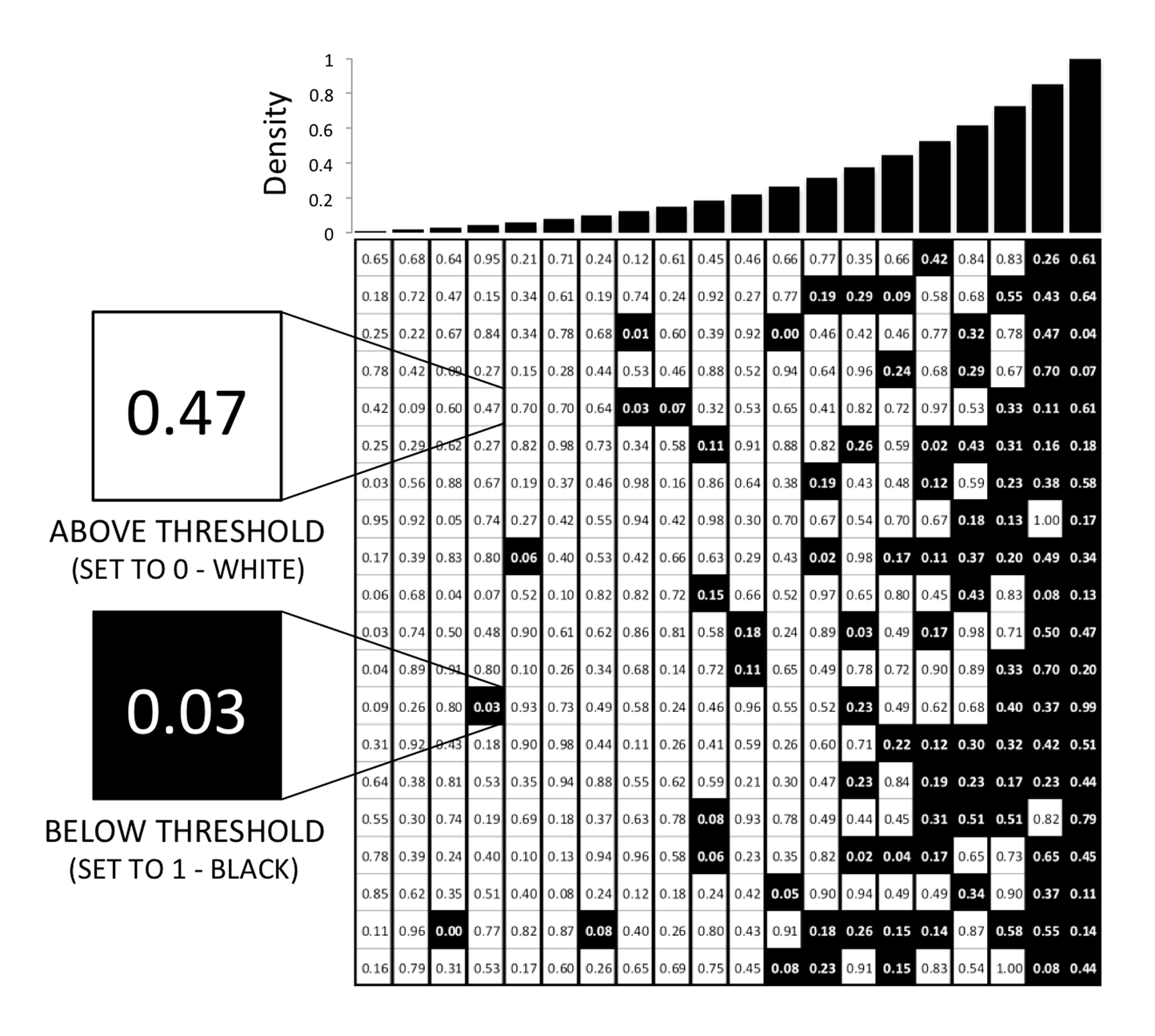

Supplement: Figure S1 — Random gradients produced by a random matrix threshold approach. A matrix of pseudo-random numbers is generated. Values greater than or equal to the density threshold are set to 0 (white), while values less than the threshold are set to 1 (black). The binary array can then be exported directly as a bitmap image file. Each nanodot is represented by one pixel. Thus, for a 400×400 µm2 sized area with 200×200 nm2 nanodots, a 2000×2000 matrix with 4 million values is required. This approach does not provide a fully random configuration since nanodots are aligned to a grid. While these patterns appear random to the eye, the underlying grid might be sensed at the cellular scale. Theoretically, it is possible to further randomize the position of dots by subdividing the area, e.g. using a 50 nm grid to position and draw 200×200 nm2 pixels, but this would come at the cost of increased computational requirements and the possibility of overlap between adjacent dots. (TIF) [file pone.0106541.s001.tif]

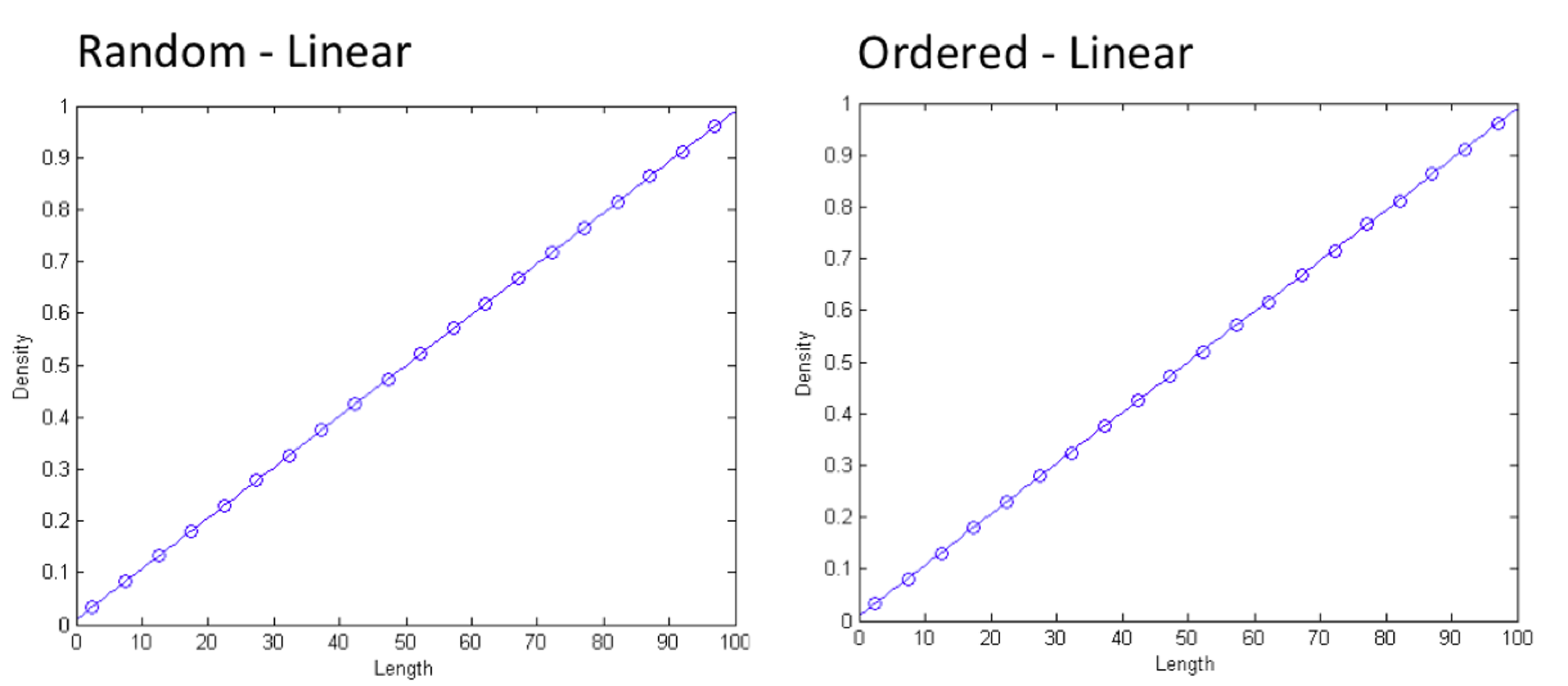

Supplement: Figure S2 — Ordered and random measured density matches programmed density over full range. Linear gradients from densities of 0.01 to 0.99 were shown to match (dots) the programed functions (line) with high fidelity for both ordered (R2 = 0.9988) and random (R2 = 1.0000) gradients. Using either approach, a high dynamic range can be achieved with near perfect match to the programmed function. (TIF) [file pone.0106541.s002.tif]

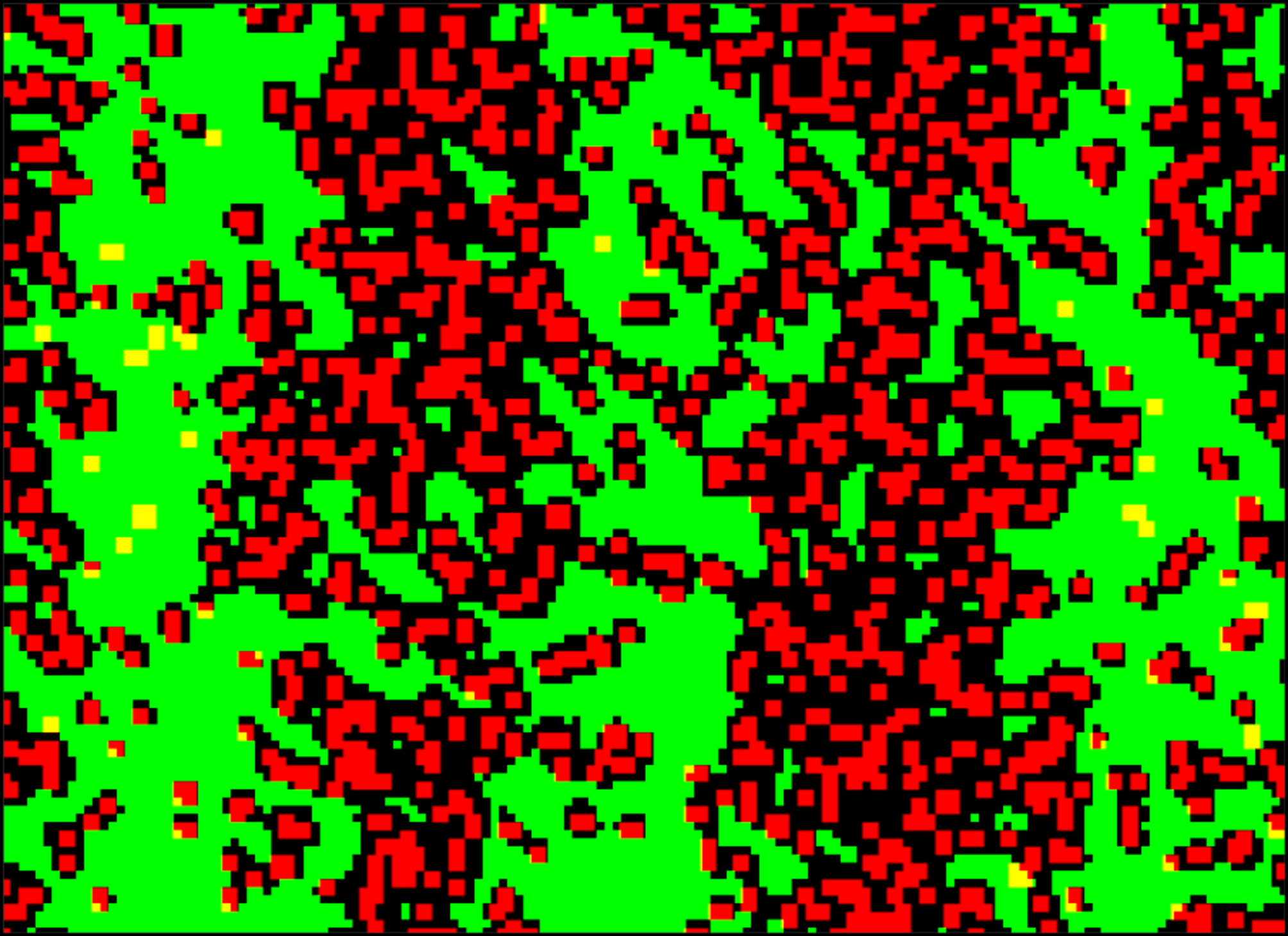

Supplement: Figure S3 — Image processing procedure to assess alignment of the DNG design and print. The fluorescent image of nanocontact printed IgG was first thresholded in ImageJ with boundary values of 31 and 255. The image was then transformed to binary and the binary values inverted to facilitate visualization. The edited fluorescent image (green) was then merged with the bitmap (red) and yellow dots, indicative of non-printed dots, were counted to determine to what extent the print matched the design. (TIF) [file pone.0106541.s003.tif]
