# Supplementary material for: Ordered, Random, Monotonic and Non-Monotonic Digital Nanodot Gradients
Source: PLoS One. 2014 Sep 5;9(9):e106541. doi: 10.1371/journal.pone.0106541 (PMC4156346; doi:10.1371/journal.pone.0106541)
Supplement: Table S1 — Function type and parameters of the one-hundred gradient array. Function type (i.e. linear, exponential, sinusoid with added trend), gradient type (ordered or random), and minimum/maximum density values for each of the 100 gradients. Gradient number is matched to density functions found in Figure 6. (PDF) [file pone.0106541.s004.pdf]

Supplementary Table 1

#	function	O/R	min (%)	max (%)		#	function	O/R	min (%)	max (%)
1	linear	O	0.02	44.44		51	sin_pluslin	R	0.02	43.96
2	linear	O	0.02	28.03		52	sin_pluslin	R	0.02	44.04
3	linear	O	0.02	19.27		53	sin_pluslin	R	0.02	43.96
4	linear	O	0.02	14.06		54	sin_pluslin	R	0.02	44.01
5	linear	O	0.02	10.71		55	sin_pluslin	R	0.02	44.08
6	linear	O	0.02	8.43		56	sin_pluslin	R	0.02	44.14
7	linear	O	0.02	6.81		57	sin_pluslin	R	0.02	44.05
8	linear	O	0.02	5.61		58	sin_pluslin	R	0.02	44.10
9	linear	O	0.02	4.70		59	sin_pluslin	R	0.02	44.15
10	linear	O	0.02	4.00		60	sin_pluslin	R	0.02	44.20
11	exponential	O	0.02	44.44		61	sin_plusexp	R	0.02	41.58
12	exponential	O	0.02	28.03		62	sin_plusexp	R	0.02	41.86
13	exponential	O	0.02	19.27		63	sin_plusexp	R	0.02	42.21
14	exponential	O	0.02	14.06		64	sin_plusexp	R	0.02	42.58
15	exponential	O	0.02	10.71		65	sin_plusexp	R	0.02	42.95
16	exponential	O	0.02	8.43		66	sin_plusexp	R	0.02	43.12
17	exponential	O	0.02	6.81		67	sin_plusexp	R	0.02	43.30
18	exponential	O	0.02	5.61		68	sin_plusexp	R	0.02	43.49
19	exponential	O	0.02	4.70		69	sin_plusexp	R	0.02	43.43
20	exponential	O	0.02	4.00		70	sin_plusexp	R	0.02	43.55
21	sin_linamp	R	0.02	43.34		71	sin_plusexp	R	0.02	43.68
22	sin_linamp	R	0.02	43.89		72	sin_plusexp	R	0.02	43.81
23	sin_linamp	R	0.02	44.17		73	sin_plusexp	R	0.02	43.68
24	sin_linamp	R	0.02	10.44		74	sin_plusexp	R	0.02	43.77
25	sin_linamp	R	0.02	10.58		75	sin_plusexp	R	0.02	43.87
26	sin_linamp	R	0.02	10.64		76	sin_plusexp	R	0.02	43.96
27	sin_linamp	R	0.02	4.82		77	sin_plusexp	R	0.02	43.83
28	sin_linamp	R	0.02	4.88		78	sin_plusexp	R	0.02	43.90
29	sin_linamp	R	0.02	4.91		79	sin_plusexp	R	0.02	43.98
30	sin_linamp	R	0.02	2.74		80	sin_plusexp	R	0.02	44.06
31	sinusoidal	R	0.02	10.02		81	linear	R	0.02	44.44
32	sinusoidal	R	0.02	15.02		82	linear	R	0.02	28.03
33	sinusoidal	R	0.02	20.02		83	linear	R	0.02	19.27
34	sinusoidal	R	0.02	15.02		84	linear	R	0.02	14.06
35	sin_pluslin_linamp	R	0.02	23.25		85	linear	R	0.02	10.71
36	sin_pluslin_linamp	R	0.02	23.69		86	linear	R	0.02	8.43
37	sin_plusexp_expamp	R	0.02	24.21		87	linear	R	0.02	6.81
38	sin_plusexp_expamp	R	0.02	24.04		88	linear	R	0.02	5.61
39	sin_plusexp_expamp	R	0.02	25.08		89	linear	R	0.02	4.70
40	sin_plusexp_expamp	R	0.02	24.99		90	linear	R	0.02	4.00
41	sin_pluslin	R	0.02	42.55		91	exponential	R	0.02	44.44
42	sin_pluslin	R	0.02	42.75		92	exponential	R	0.02	28.03
43	sin_pluslin	R	0.02	42.99		93	exponential	R	0.02	19.27
44	sin_pluslin	R	0.02	43.23		94	exponential	R	0.02	14.06
45	sin_pluslin	R	0.02	43.48		95	exponential	R	0.02	10.71
46	sin_pluslin	R	0.02	43.59		96	exponential	R	0.02	8.43
47	sin_pluslin	R	0.02	43.71		97	exponential	R	0.02	6.81
48	sin_pluslin	R	0.02	43.83		98	exponential	R	0.02	5.61
49	sin_pluslin	R	0.02	43.80		99	exponential	R	0.02	4.70
50	sin_pluslin	R	0.02	43.87		100	exponential	R	0.02	4.00

Legend

O/R	Ordered/Random
sin_linamp or sin_expamp	Sinusoid with linearly or exponentially increasing amplitude
sin_pluslin or sin_plusexp	Sinusoid plus a linear or exponential average trend
sin_pluslin_linamp	Sinusoid with linearly increasing amplitude and linear average trend
sin_plusexp_expamp	Sinusoid with exponentially increasing amplitude and exponential average trend
